# Association of NOS3 (rs 2070744) and SOD2Val16Ala (rs4880) gene polymorphisms with increased risk of ESRD among Egyptian patients

**DOI:** 10.1186/s43141-021-00260-w

**Published:** 2021-10-18

**Authors:** Afaf Elsaid, Omnia Samir eid, Samy B. Said, Rasha F. Zahran

**Affiliations:** 1 Genetics Unit, Children Hospital, Mansoura University, Mansoura, Egypt; 2 Department of Chemistry, Biochemistry Division, Faculty of Science, Damietta University, New-Damietta, Egypt; 3 Department of Chemistry, Faculty of Science, Damietta University, New-Damietta, 34517 Egypt

**Keywords:** Chronic kidney failure, End-stage renal disease, Gene polymorphisms, NOS3 rs 2070744, SOD2 Val16Ala (rs4880)

## Abstract

**Background:**

Chronic kidney Failure (CKD), particularly End-Stage Renal Disease (ESRD), may be serious ill-health related to a high death rate. Uremic syndrome leads to increased oxidative stress, inflammation, and dyslipidemia. Our study aimed at identifying the association of NOS3 (rs 2070744) and SOD2 Val16Ala (rs4880) gene polymorphisms within ESRD Egyptian patients.

**Methods:**

This work was conducted on 100 ESRD and 16 CKD Egyptian patients who were compared to 100 healthy controls. DNA was genotyped for these variants using the (T-ARMS-PCR) technique.

**Results:**

ESRD patients showed a significant association of the genotype of NOS3 gene polymorphism compared with healthy controls (*P* = 0.032). In the contrast, the present study revealed that no statistically significant differences were found among the CKD, ESRD, and control groups as regards the SOD2 genotypes (*P* = 0.064).

**Conclusions:**

Our findings indicated a significant association between NOS3 (rs 2070744) gene polymorphism and increased risk of ESRD and CKD among Egyptian patients.

## Background

Chronic kidney disease (CKD) is becoming a major public health problem worldwide. CKD is defined as a progressive loss of renal function, measured by a decline in glomerular filtration rate (GFR < 60 mL/min/1.73 m^2^), which is characteristically accompanied by irreversible pathological alterations within the kidney. This pathology has a complicated interrelationship with other diseases. Diabetes (DM) and hypertension (HT) are the main risk factors for CKD, and CKD is also associated with cardiovascular morbidity and mortality, even in early stages and in young patients [1].

With 850 million individuals affected by kidney diseases, CKD is now considered a major global public priority [2].

Chronic kidney disease is a main global health problem with an increasing prevalence in Egypt. By 2040, it is expected that CKD will have become the 5th leading reason for death, this increasing CKD burden is driven in part by the aging population structure (CKD is ~ 8× more common in adults > 70 years old compared to persons < 40 years of age) [3].

Chronic kidney disease is a complex heterogeneous disease, with contributions from both genomic and environmental factors. CKD heritability has been estimated to be high (30–75%) [4].

CKD patients have many affected physiological pathways. Variations in the genes regulating these pathways might affect the incidence and predisposition to this disease [1].

Several hundred genes are currently known to contain mutations that can cause single-gene disorders with a kidney phenotype, as well as dozens of genetic loci in which common genetic variants are associated with kidney function in the normal range and with complex kidney diseases [5].

Most cases of CKD are caused by chronic-degenerative diseases and endothelial dysfunction is one of the reasons that contribute to its pathophysiology. One of the most imperative mechanisms for the suitable functioning of the endothelium is the regulation of the synthesis of nitric oxide [6].

Nitric oxide (NO), the most dilator discharged by the epithelial tissue, is synthesized by the chemical reaction of l-arginine through the action of the membrane-associated epithelium organic isoform of NO synthase (ecNOS). A reduced expression of eNOS might contribute to the epithelium pathology in nephritis [7].

NOS3 (eNOS) gene has 23.605 bases and is located at 7q36.1. It has twenty-six exons and codes for NOS3, an enzyme composed of 1203 amino acids with an Mwt. of 133.289 Da [8].

The eNOS factor has 2 common alleles comprise of 4 repeats (a) and 5 repeats (b) that manufacture 2 homozygous (aa and bb) and 1 heterozygous (ab) genotypes [9].

The NOS3 gene polymorphisms 894G>T or Glu298Asp (rs1799983), 27-bp repeat in intron 4 (VNTR) 4b/a variations, and − 786 T>C have all been explored in numerous disorders linked to diabetic nephropathy in diverse publications [10].

The functional effect of these variants is to reduce the expression of mRNA or change the function of eNOS, resulting in a reduction in NO production. Genetic variations in the eNOS gene have an essential role in the development of renal disease [11].

Two alleles have been found in the 27 bp VNTR of intron 4, the larger of which has five tandem 27 bp repeats and the smaller of which has four 27 bp repeats. They dubbed these two alleles the “4b” and “4a” alleles, respectively, to describe the wild-type and mutant alleles. The impact of this polymorphism on eNOS expression and activity is poorly defined [12].

Oxidative stress is linked to inflammation, cardiovascular disease, epithelium pathology, and CKD progression [13].

Redox state in mitochondria is regulated by not only Mn-SOD but also other antioxidative enzymes, including Cu/Zn-SOD, GPx, and the PRx/TRx system. However, as Mn-SOD catalyzes the first step in ROS scavenging in mitochondria, treatments targeted at supporting Mn-SOD integrity and function may lead to effective treatments to prevent the onset and progression of kidney disease [14].

Metal SOD (MnSOD) is one of the crucial enzymes that defend against ROS within the mitochondria. The SOD2 gene is set on the body at 6q25.3. Currently, many single-nucleotide polymorphisms (SNPs) within the MnSOD cistron are rumored, of that the foremost extensively studied one is Val16Ala [15].

**Fig. 1:**
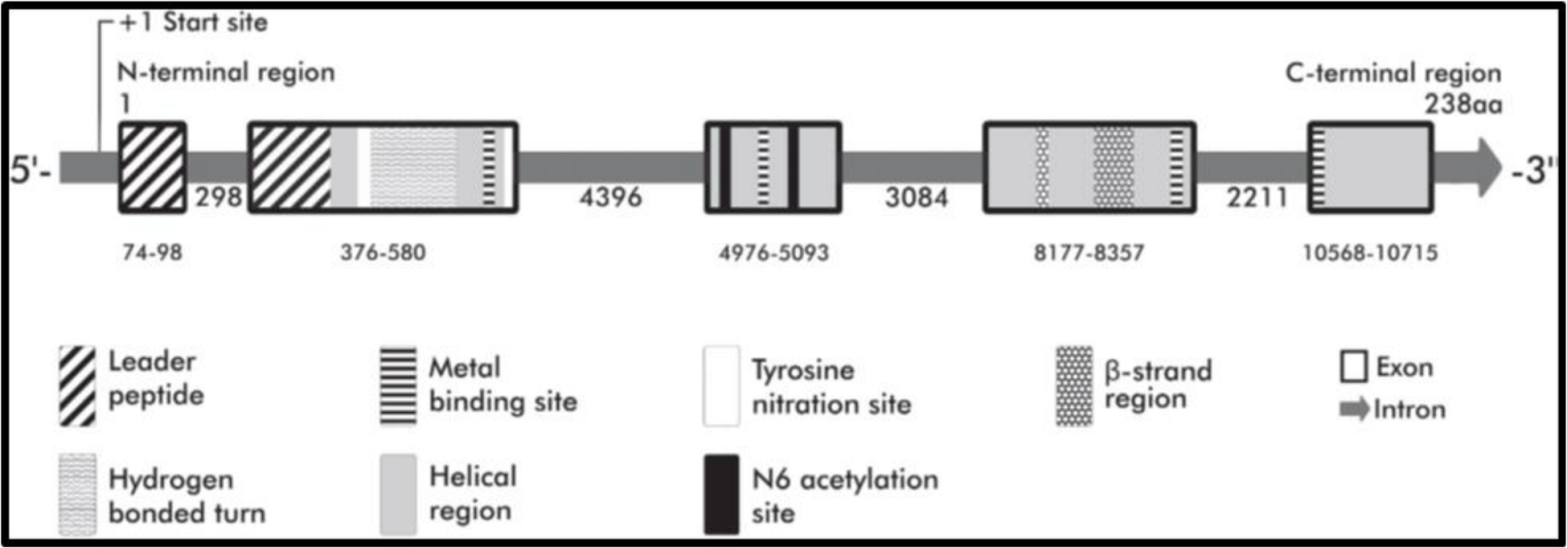
Genomic organization of the human MnSOD gene [16]

SOD2 (MnSOD) is a single-copy gene having five exons and four introns separated by normal splice junctions. The start site is used to illustrate the position and size of each exon in terms of DNA base pairs, as shown in Fig. 1, Exon locations are aligned to highlight the gene’s domain organization, adapted from Dhar and St. Clair [16].

The genetic variations that encode antioxidant enzymes were associated with creatinine levels, glomerular filtration rate (GFR), albumin and phosphorus levels, erythropoietin resistance index, C-reactive protein, and ferritin levels which play a serious role in CKD progression [1].

Therefore, the purpose of this research is to see how NOS3 (rs 2070744) and SOD2 Val16Ala (rs4880) gene polymorphisms affect CKD progression in Egyptians.

## Methods

### Ethical recognition of research

Before being included in the study, all individuals gave written informed consent, and blood samples were taken according to protocols approved by the hospital’s Outpatient Clinic’s Ethics Committee.

### Study populations

Patients with CKD were screened and diagnosed between September 2018 and March 2019 from the Outpatient Clinic of the hospital. The study involved a total of 216 Egyptian adults, including 116 patients suffering kidney pathologies at different stages, and 100 controls. The participants were divided as follow:

1. ESRD group: this group included 100 females and males ESRD patients whose ages ranged from 18 to 82 years old (mean age = 47 years and SD = 16.1).
2. CKD group: this group included 16 females and males CKD patients whose ages ranged from 15 to 58 years old (mean age =40 years and SD = 11.09). The clinical data of all patients were obtained from medical records. Patient's data included age, sex, and laboratory investigation such as Hb conc., creat conc., bilirubin, albumin, SGPT, SGOT, FBS, HBS Ag, and HCV.
3. Control group: this group included 100 healthy samples (54 females and 46 males) for comparison; their age and sex were matched with the chronic kidney disease patients, and their families did not have any record of chronic kidney disease history (mean age = 37 years, SD = 12.7).

### Sampling

Three milliliters of peripheral blood was collected from all subjects using vacationers and transferred to test tubes containing EDTA solution as an anticoagulant to obtain whole blood, for extraction and purification of DNA. Peripheral blood samples are also utilized to investigate biochemical markers related to CKD.

### DNA extraction and purification

The handling and extraction of genomic DNA for all participants enrolled in this work was accomplished by using the generation DNA purification capture column kit (BioFlux, China). The extraction procedures were carried out based on the prescripts provided by the producer for the peripheral blood samples.

DNA in the sample was released using proteinase K (PK) solution and lysis B buffer. The released DNA was bound exclusively and specifically to the biospin membrane in the presence of lysis B buffer and ethanol under the appropriate salt iron and pH conditions. Denatured protein and other contaminants were removed with twice washing procedures. With the elution buffer, the DNA was then eluted from the membrane [17].

The amplification and genotyping of eNOS (rs 2070744) and MnSOD Val16Ala (rs4880) Gene variants were described using the tetra-primer amplification refractory mutation system polymerase chain reaction (T-ARMS-PCR) [18].

### Genetic analysis

#### Determination of MnSOD genotype

T-ARMS-PCR was performed by using the tetra primer (forward inner primer, reverse inner primer, forward outer primer, reverse outer primer) and the amplicons purification using QIAquick PCR purification kit (Qiagen) [19].

The DNA was amplified using specific oligonucleotide primers based on the published sequence (Biolegio, Nijmegen-The Netherlands). Two primer pairs were used to accentuate and designate the genotype of a deoxyribonucleic acid fragment, as well as the Ala16Val SNP within the human MnSOD sequence. The 3′-end of the allele-specific primers were underlined

F1 (forward) 5′-CACCAGCACTAGCAGCATGT-3′;

F2 (forward)5′GCAGGCAGCTGGCTaCGGT-3′;

R1 (reverse) 5′-ACGCCTCCTGGTACTTCTCC-3′

and R2 (reverse)5′-CCTGGAGCCCAGATACCCtAAAG-3.

PCR was conducted in an exceedingly total volume of 25 μL containing 4 μL of genomic DNA because the templet, 4 μL of every primer, 13 μL Master Mix (2×). PCR amplification was conducted with primary denaturation at 95 °C for 2 min, followed by 35 cycles of 1 min of denaturation at 94 °C, 1 min of hardening at 60 °C, 1 min of extension at 72 °C, and a further a pair of min extension at 72 °C at the tip of the ultimate cycle. Next, The PCR products were electrophoresed on 2% agarose gel and visualized using ethidium bromide under ultraviolet illumination below ultraviolet radiation.

**Fig. 2:**
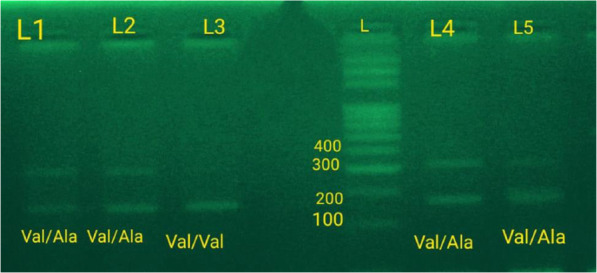
Genotyping of the SOD2 gene (L = 100 bp ladder DNA marker) (Val/Val: homozygous wild-type and Val/Ala: heterozygous-genotype). (L=lane)

The PCR product for MnSOD cistron was detected at 189 bp for wild-type (Val/Val), and 366 bp for mutant type (Ala/Ala) then photographed by the photographic camera. Once the sample had a pair of bands at 189 bp in the wild tube and 366 bp within the mutant tube, it meant it had Val/Ala heterozygote genotyping. On the opposite hand, once the sample had a band that appeared at 189 bp in the wild-type tube and no band within the mutant factor, this meant it had Val/Val homozygote genotyping. Once the sample had a band at 366 bp within the mutant factor and no band in the wild-type factor, this meant it had Ala/Ala homozygote genotyping as shown in Fig. 2.

#### Determination of eNOS genotype

PCR was performed by using only one pair of primers, 2 oligonucleotide primers that flank the region of the 27-bp repeat sequence in intron 4 of the eNOS gene were used for polymerase chain reaction (PCR) amplification. The eNOS primers were synthesized by Integrated DNA Technologies, Inc. (IDT, Santa Clara, CA, USA). The forward primer was 5′-AGGCCCTATGGTAGTGCCTTT-3′ and the reverse primer was 5′-TCTCTTAGTGCTGTGGTCAC-3′ [20].

PCR was conducted in an exceedingly total volume of 25 μL in only one tube containing 4 μL of genomic DNA, 4 μL of every primer, 13 μL Master Mix (2×). The reaction mixture was heated to 95°C for 6 min for denaturation and then subjected to 35 cycles at 95 °C for 1 min, annealing at 58 °C for 1 min and extension at 72 °C for 2 min, followed by a final extension step at 72 °C for 7 min.

The PCR products were electrophoresed on 2% agarose gel and visualized using ethidium bromide under ultraviolet illumination. The PCR product for the eNOS gene (intron 4a/4b) was detected at 420 bp for wild-type (4b) and 393 bp for mutant-type (4a) then photographed by the digital camera.

**Fig. 3:**
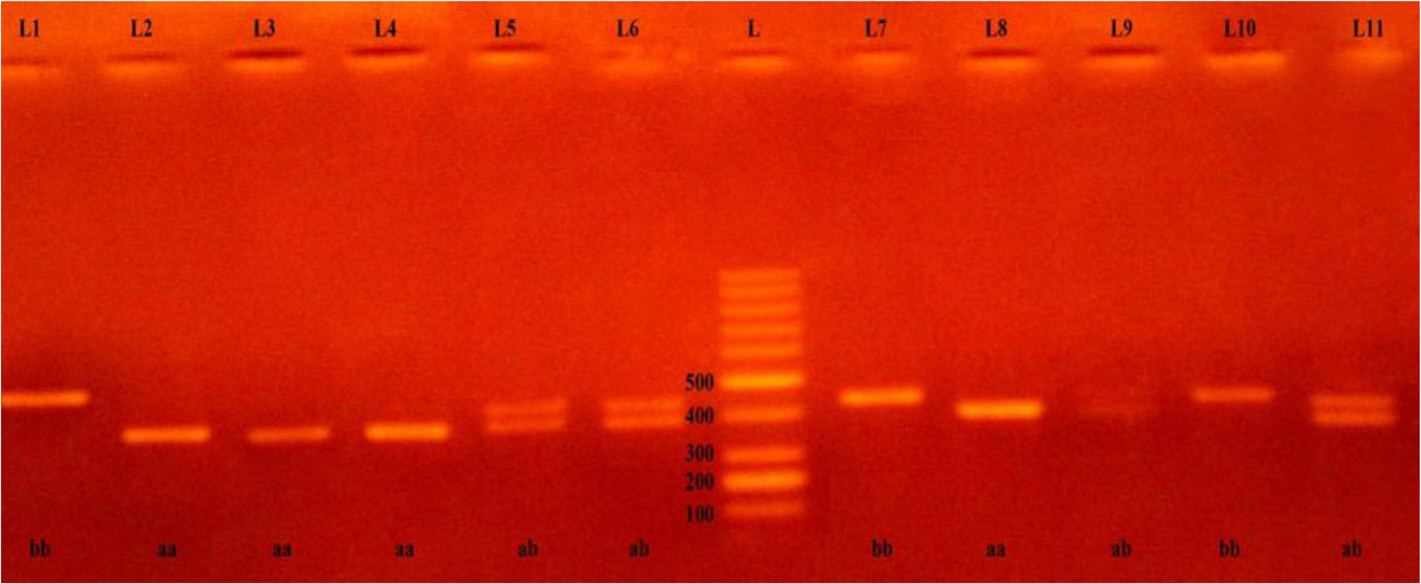
Genotyping of the VNTR in intron 4 of eNOS gene (L = 100 bp ladder DNA marker) (bb: homozygous wild-type, ab: heterozygous-genotype and aa: homozygous-genotype). (L=lane)

When the sample had 2 bands at 420 bp wild-type 4b allele and 393 bp for mutant-type it meant it had 4ab heterozygote genotyping. On the other hand, when the sample had a band at 393 bp for mutant-type and no band at 420 bp for wild-type, this meant it had 4aa homozygote genotyping. When the sample had a band at 420 bp for wild-type and no band for mutant-type, this meant it had 4bb homozygote genotyping as shown in Fig. 3.

### Statistical analysis

Data were collected, coded, revised, and entered into the Statistical Package for science (IBM SPSS) version 20. For qualitative data, mean, standard deviations, and ranges were used; for quantitative data with a parametric distribution, median with interquartile range (IQR) was used; and for quantitative data with a non-parametric distribution, median with interquartile range (IQR) was used.

When comparing two groups with qualitative data, the chi-square test was utilized, and the Fisher exact test was used instead of the chi-square test when the expected count in any cell was less than 5. The independent *t* test was used to compare two groups with quantitative data and a parametric distribution, while the Mann-Whitney test was used to compare 2 groups with qualitative data and a non-parametric distribution.

The one way analysis of variance (ANOVA) test was used to compare more than two groups with quantitative data and parametric distribution, and the Kruskal-Wallis test was used to compare more than 2 groups with quantitative data and non-parametric distribution.

**Fig. 4:**
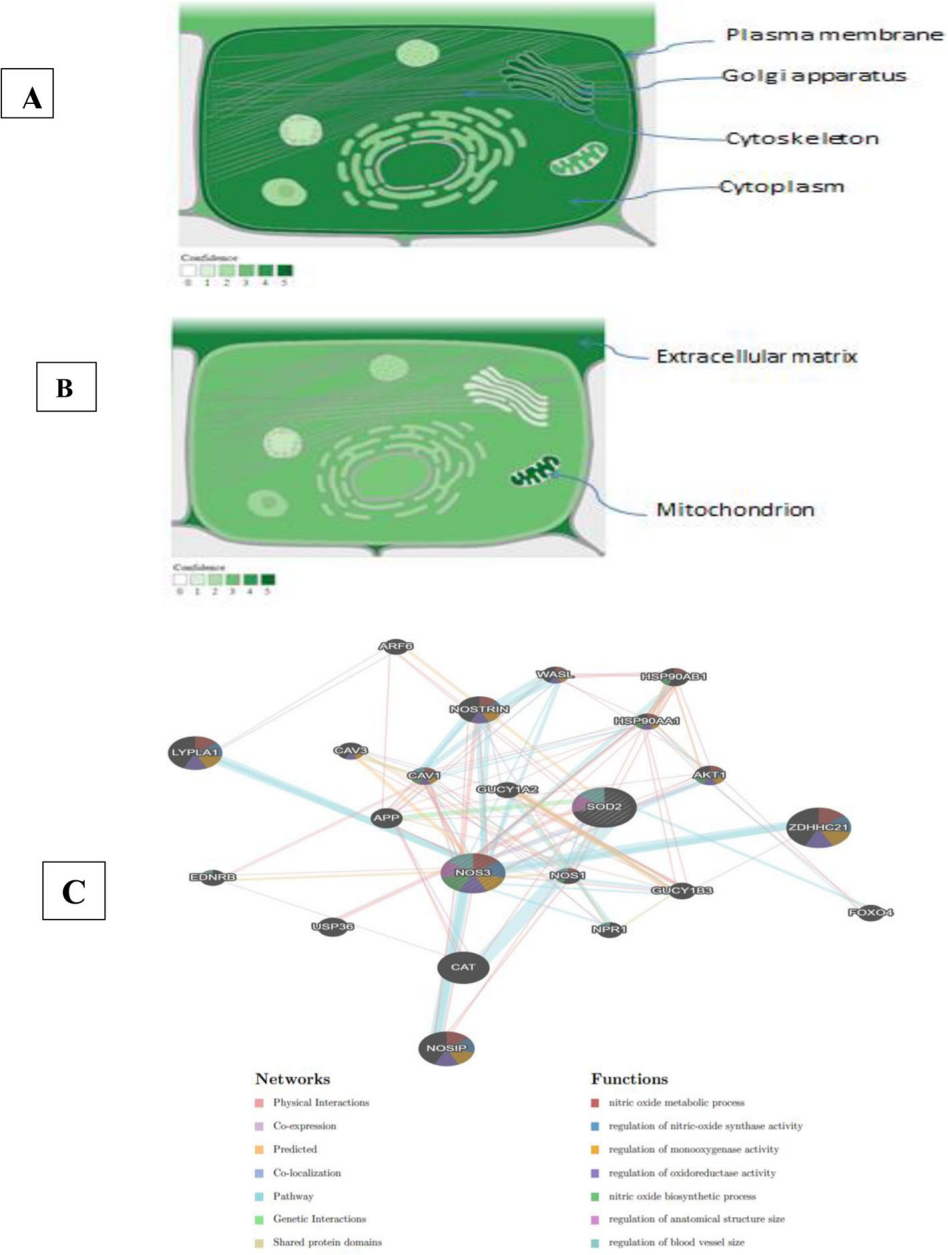
In silico data analysis. **A** Subcellular localization of NOS3protein. **B** Subcellular localization of SOD2 protein. Darker color, according to the provided color key, is indicating more abundance (data source: https://compartments.jensenlab.org/Search). **C** Gene-gene functional interaction network generated by Gene MANIA. The network nodes have been colored by function (i.e., gene ontology annotation) (data source: https://genemania.org/search/homo-sapiens/SOD2/NOS3)

The margin of error acceptable was set to five because the confidence interval was set at 95%. Because of the following, the *P* value was deemed significant: *P* > 0.05: non-significant (NS), *P* < 0.05: significant (S), *P* < 0.01: highly significant (HS). Figure 4 illustrates in silico data analysis.

## Results

### The clinical characteristics of all participants in the study

The genotypes of 216 individuals (100 ESRD patients, 16 CKD patients, and 100 healthy controls) and the essential clinical and demographic characteristics of CKD patients and unrelated healthy controls are described in Table 1. Our findings indicated that there was a statistically significant difference between CKD, ESRD, and control group regarding the gender with a statistically significant increase in male percent in ESRD group in comparison to CKD and control group.

**Table 1:** Clinical characteristics of studied subjects

		CKD (***n*** = 16)	ESRD (***n*** = 100)	Control (***n*** = 100)	***P*** value
**Gender**					0.004
**Female**		8 (50%)	31 (31%)	54 (54%)	
**Male**		8 (50%)	69 (96%)	46 (46%)	
		**Mean ± SD**	**Mean ± SD**	**Mean ± SD**	
**Age**		40.88 ± 11.10	47.10 ± 16.11	37.84 ± 12.73	< 0.001
**HB conc.**		16.04 ± 24.54	10.10 ± 1.96	12.31 ± 1.26	0.002
**Creatinine conc.**		3.69 ± 5.16	8.08 ± 3.03	0.82 ± 0.20	< 0.001
**Bilirubin**		0.47 ± 0.14	0.63 ± 0.20	0.40 ± 0.13	< 0.001
**SGPT**		30.0 ± 4.40	27.06 ± 21.85	21.06 ± 8.61	0.012
**SGOT**		38.63 ± 3.70	30.40 ± 21.19	29.38 **±** 9.01	0.094
**Ca**		–	9.18 ± 0.80	8.27 **±** 1.14	< 0.001
**P**		–	3.34 ± 0.58	5.98 **±** 2.40	< 0.001
**PTH**		–	31.30 ± 11.3	477.54 **±** 439.73	< 0.001
**HBs Ag**	**Negative**	16 (100.0%)	100 (100.0%)	0 (0.0%)	NA
**HCV**	**Negative**	16 (100%)	74 (74%)	100(100%)	< 0.001
	**Positive**	0 (0%)	26 (26%)	0 (0%)	
**FBS**		106.56 ± 43.58	125.5 ± 67.59		0.279
**Urea**		107.75 ± 30.36			
**GFR**		28.75 ± 13.91			

Also, a highly statistically significant difference was found when comparing age between CKD, ESRD, and control group *P* < 0.001. Also, there was a highly significant increase in Hb, creatinine, bilirubin, and SGPT in the CKD and ESRD groups in comparison to the control group. Moreover, ESRD patients had  a highly significant increase in Ca, Phosphate, and PTH in comparison to the control group (Table 1).

### Association of MnSOD Val16Ala (rs4880) polymorphism with CKD

The Val16Ala polymorphism in MnSOD was found to be in Hardy–Weinberg equilibrium. The Ala allele was found 18% in control patients, 31.2% in CKD cases, and 26% in ESRD cases (*p* = 0.064). The genotypes of the groups were determined by PCR. Fragments containing 366 bp (Ala) and 189 bp (Val). The genotype distribution in patients and controls is shown in Table 2 and Fig. 2.

**Table 2:** Distribution of MnSOD Val16Ala polymorphism in cases and controls

		CKD (***n*** = 16)		ESRD (***n*** = 100)		Control (***n*** = 100)		Chi-square test	
		No.	%	No.	%	No.	%	*χ*2	***P*** value
**Genotype SOD**	**Ala/ala**	2	12.5	10	10	10	10	**8.851**	**0.064**
	**Val/ala**	6	37.5	32	32	16	16		
	**Val/Val**	8	50	58	58	74	74		
	**Val**	22	68.8	148	74	164	82		
	**Ala**	10	31.2	52	26	36	18		

This study shows that there was no statistically significant difference between clinical data and genotype SOD in CKD where *P* > 0.05 and shows that there was a statistically significant increase in Val/Val with ca conc. in ESRD where *P* = 0.047 as shown in Table 3.

**Table 3:** Comparison between genotype SOD in ESRD among clinical data

		Genotype SOD						One way ANOVA	
		Ala/Ala		Val/Ala		Val/Val			
								***F***	***P***
**Hb Conc.**		10.21	1.61	10.36	1.82	9.93	2.05	0.524	0.594
**Duration of dialysis (months)**		10.40	8.25	20.00	25.96	40.74	42.06	5.398	0.006
**Ca Conc.**		7.65	1.78	8.07	1.12	8.49	0.97	3.150	0.047
**Phosphate**		4.85	1.43	6.20	2.39	6.04	2.51	1.277	0.283
**PTH**		449.20	318.01	439.66	380.49	503.33	489.40	0.236	0.791
**Creat Conc.**		8.07	3.39	8.00	3.52	8.12	2.71	0.016	0.984
**Bilirubin**		0.67	0.15	0.61	0.22	0.63	0.19	0.397	0.673
**Albumin**		3.99	0.33	3.85	0.46	3.80	0.43	0.846	0.432
**SGPT**		19.90	6.51	29.94	22.03	26.71	23.34	0.819	0.444
**SGOT**		21.70	4.03	33.38	21.26	30.26	22.65	1.164	0.317
**FBS**		156.70	80.88	106.03	39.87	131.00	75.82	2.672	0.074
		No	%	No	%	No	%	Chi-square test	
**HBsAg**	**Negative**	10	100.00	2	100.00	30	100.00	NA	NA
**HCV**	**Negative**	10	100.00	2	100.00	20	66.70	5.131	0.162
	**Positive**	0	0.00	0	0.00	10	33.30		

### Association of eNOS gene polymorphism with increased risk of CKD

The genotypes of the groups were determined by PCR. Fragments containing 420 bp (eNOS b allele) and 393 bp (eNOS a allele). The genotype distribution in patients and controls is shown in Fig. 3.

**Fig. 5:**
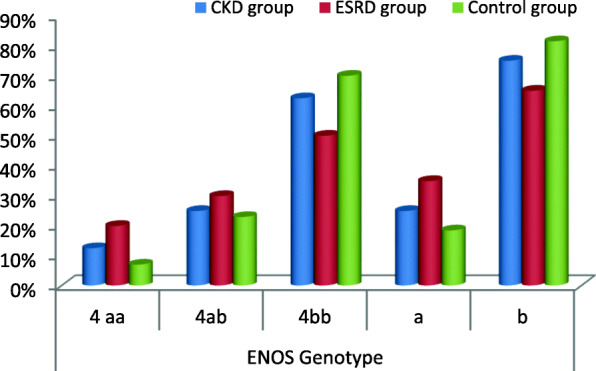
NOS3 regarding CKD, ESRD, and control group

We found that there was a statistically significant difference between CKD, ESRD, and control group among eNOS genotyping where *P* = 0.032. This study shows that the a allele has a higher statistically significant difference between CKD, ESRD, and control group among eNOS Genotyping than b allele as shown in Table 4, and NOS3 regarding CKD, ESRD, and control group is shown in Fig. 5.

**Table 4:** Distribution of eNOS intron 4 polymorphism in cases and controls

		CKD group (***n*** = 16)		ESRD group (***n*** = 100)		Control group (***n*** = 100)		Chi-square test	
		No	%	No	%	No	%	*χ*2	*P* value
**eNOS** **Genotype**	**4aa**	2	12.5	20	20	7	7	10.58	0.032
	**4ab**	4	25	30	30	23	23		
	**4bb**	10	62.5	50	50	70	70		
	**a**	8	25	70	35	37	18.5		
	**b**	24	75	130	65	163	81.5		

OR (95% CI) (1.7-4.1)

In our study, we found that there was a statistically significant increase in 4aa in comparison to 4ab and 4bb with phosphate and bilirubin in ESRD by comparison between eNOS genotype in ESRD among clinical data as shown in Table 5.

**Table 5:** Comparison between eNOS genotype in ESRD among clinical data

		eNOS genotype						One-way ANOVA	
		4aa		4ab		4bb			
		Mean	SD	Mean	SD	Mean	SD	***F***	***P***
**Hb Conc.**		9.67	2.17	10.27	1.42	10.16	2.16	0.609	0.546
**Duration of dialysis (months)**		39.05	44.30	28.40	35.73	29.48	35.01	0.583	0.56
**Ca Conc.**		7.94	0.69	8.38	1.22	8.33	1.23	1.051	0.354
**Phosphate**		6.42	2.32	4.95	1.68	6.41	2.63	4.176	0.018
**PTH**		466.20	397.92	397.70	249.17	529.98	534.57	0.854	0.429
**Creat Conc.**		7.65	2.67	9.08	3.13	7.64	3.01	2.428	0.094
**Bilirubin**		0.72	0.16	0.48	0.17	0.69	0.18	15.595	<0.001
**Albumin**		4.01	0.33	3.79	0.44	3.79	0.46	1.962	0.146
**SGPT**		19.20	7.36	24.33	16.47	31.84	27.02	2.824	0.064
**SGOT**		21.55	5.79	32.10	17.06	32.92	26.10	2.251	0.111
**FBS**		109.40	55.48	110.93	54.26	140.84	76.28	2.636	0.077
		**No**	**%**	**No**	**%**	**No**	**%**	**Chi-square test**	
**HBsAg**	**Negative**	20	100.00	30	100.00	50	100.00	NA	NA
**HCV**	**Negative**	18	90.00	21	70.00	35	70.00		
	**Positive**	2	10.00	9	30.00	15	30.00	3.326	0.190

## Discussion

The chronic renal disorder could be a variety of renal disorders during which there's gradual loss of excretory organ performance over several months to years. Initially, there are generally no symptoms; later, symptoms could embody leg swelling, feeling tired, vomiting, loss of craving, and confusion [21].

To our knowledge, this is the first study to interpret the association of the MnSOD Val16Ala and NOS3(rs 2070744) gene polymorphisms as a risk factor in Egyptian patients who had chronic kidney disease. The present study revealed a highly statistically significant difference (*P* = 0.004) among the study groups as regards gender as females represented 50%, 31%, 54% of the CKD, ESRD, and control groups respectively whereas males represented 50%, 69%, 46% of the CKD, ESRD and control groups respectively. Furthermore, a very highly statistically significant difference (*P* < 0.001) was found among the study groups as regards age which was mainly between the ESRD group, the gender distribution revealed in the current study comes in line with what was published by Goldberg and Krause in 2016 as they noted that while the prevalence of CKD tends to be higher in women, the disease is more severe in men, who also have a higher prevalence of ESRD. As regards the mean age of patients revealed in this study, it agrees with what was published by the Centers for Disease Control and prevention in 2020 as they mentioned that after the age of 40, kidney filtration begins to decline at a rate of about 1% per year. They went on to say that, in addition to natural renal aging, several illnesses that harm the kidneys, such as diabetes, high blood pressure, and heart disease, are more common in older persons.

The Val16Ala (rs4880) SOD2 gene polymorphism is the greatest investigated for disorders associated with CKD and its renal consequences. The SOD2 Val16Ala variations impair the antioxidant enzyme's processing efficiency by producing conformational changes that result in decreasing the efficiency of mitochondrial transit of the Val form, which can diminish MnSOD activity and level in the mitochondria and contribute to redox reactions [22].

In terms of SOD genotypes, no statistically significant differences were detected between the CKD, ESRD, and control groups in this investigation. Furthermore, the current investigation discovered no statistically significant differences between the different SOD genotypes in the CKD group as regards the mean levels of creatinine, bilirubin, albumin, SGPT, SGOT, and FBS. Meanwhile, numerous studies have shown significant associations between the SOD2 gene Val16Ala polymorphism and albuminuria as in the studies published by Yang et al. [23]; Liu et al. [24]; Ascencio-Montiel et al. [25] as well as in the meta-analysis published by Tian et al. [26]. The difference between results might be attributed to the different criteria of the included patients as these studies examined the association in type 2 diabetic patients meanwhile, the proportion of diabetics in the patient's group in the current study was only 25%. Furthermore, differences in the disease status and ethnic backgrounds might have contributed to this difference.

Moreover, our study also revealed that no statistically significant differences were found between the different SOD genotypes as regards the prevalence of hypertension or diabetes. On the contrary, Val/Val genotype was found to be more common in both type 1 and type 2 diabetes patients by Flekac et al. [27], which was performed on 120 types 1 diabetes, 306 types 2 diabetes, and a control group of 140 healthy subjects. The difference between results might be explained by the small number of diabetic patients enrolled in the current study which was not enough to illustrate the association between the SOD genotypes and diabetes. Furthermore, many environmental factors such as dietary and plasma antioxidant capacity as significant modifying factors have been studied concerning diabetes and some of its complications, and study results have shown that these antioxidant statuses can mediate oxidative stress consequences in patients ([23, 28].

Genetic polymorphisms in the eNOS gene are shown to affect their activity and may also promote CKD progression in diabetic and hypertension patients [11].

The current study revealed a highly statistically difference (*P* = 0.032) between the patient and control groups as regards eNOS genotype with a higher percentage of those having 4aa and 4ab genotypes being in the patient’s group and a higher percentage of those having 4bb genotype being in the control group. eNOS gene is known as a candidate gene in cardiovascular and renal diseases [29]. A higher incidence of the 4a allele was also observed among Czech patients with ESRD in the study published by Merta et al. [30], who performed their study on 128 Czech patients with ADPKD and 93 patients with IgA nephropathy in addition to 100 genetically unrelated healthy subjects. Similar results were achieved by Elshamaa et al. [31], who performed their study on 78 children with CKD and 30 healthy controls as they found a significantly higher frequency of the aa genotype and eNOS 4a allele carriers among CKD children than in controls. Similarly, the 4a allele was significantly associated with CKD advancement in the study published by Elumalai et al. [32], who performed their study on 53 ADPKD patients and 94 unrelated healthy controls. On the other hand, the frequencies of aa and ab genotypes in the patient populations were not significantly different from those observed in the control group in the study published by Lamnissou et al. [33], who performed their study on 361 ESRD patients and 295 healthy subjects from Greece and Cyprus. However, they found that there was faster progression to ESRD in the group of ADPKD patients who carried allele a.

NO plays an important role in cell growth and renal blood vessels. It is well known that NO acts as a vasodilator. In addition, NO can also inhibit stromal growth and mesangial cell production. As we age, reduced levels of NO can cause renal vasoconstriction, sodium retention, increased matrix production, and mesangial fibrosis. Also, the concentration of NO subtypes observed in the bone marrow area is higher than in other areas. On the other hand, NO isoforms are reduced in the renal cortex. Therefore, they cause a decrease in blood flow in the renal cortex of the elderly and this was revealed in our study when compared to age between CKD, ESRD, and control group *P* < 0.001.

Also, the current study similar to the study published in 2021 by Gunawan et al. They found that the 4a allele was significantly associated with CKD advancement [34].

In contradiction to the previous findings, the study published by Deepashree et al. in 2021, did not observe any association between the NOS3 gene polymorphism and nephropathy. I think the differences in the disease status and ethnic backgrounds might have contributed to this difference because they performed their study on 253 Type 2 diabetes with nephropathy patients (DN) and 104 healthy subjects [35].

## Conclusions

This is the first study that explores a significant association with NOS3 (rs 2070744) gene polymorphism within the increased risk of ESRD and CKD among Egyptian patients. However, some research did not detect a link; therefore, the findings are debatable. More research in different populations is needed to find potential genetic risk factors for CKD.

## Data Availability

The authors declare that all generated and analyzed data are included in the article.

## Abbreviations

ADPKD: Autosomal dominant polycystic kidney disease
CKD: Chronic kidney disease
Cu/Zn-SOD: Copper/zinc superoxide dismutase
DM: Diabetes mellitus
EDTA: Ethylene diamine tetraacetic acid
eNOS: Endothelial nitric oxide synthase
ESRD: End-stage renal disease
FBS: Fasting blood sugar
GFR: Glomerular filtration rate
GPx: Glutathione peroxidase
HTN: Hypertension
MnSOD: Manganese superoxide dismutase
PTH: Parathyroid hormone
PRx/TRx: Peroxiredoxin/thioredoxin system
PCR: Polymerase chain reaction
ROS: Reactive oxygen species
SNPs: Single-nucleotide polymorphisms
VNTR: Variable number tandem repeat
